# Medico-Legal Trials in Lunacy

**Published:** 1863-04

**Authors:** 


### MEDICO-LEGAL TRIALS IN LUNACY.

### THE BRIDPORT MURDER.

Charles Fooks, forty-nine, farmer, was placed at the bar, charged with the wilful murder of Daniel Joseph Stone, at Walditch, on the 29th August, 1862.

Mr. Collier, Q.C., said the prisoner was a farmer occupying a good position in his parish. The deceased was the first cousin of the prisoner, and a near neighbour. These near relatives and neighbours could not agree, the prisoner showing a violent feeling of animosity against his cousin. On the morning of the 29th, the prisoner was standing outside his door with a gun in his hand. The deceased passed by within a short distance of where he was standing. He put the gun to his shoulder, took deliberate aim, and shot him at the back of the head. The prisoner returned to his own house, went upstairs, and locked himself in his bedroom, and a few minutes after another report of a gun was heard from where he was. The door was broken open, and he was found on the floor, sensible, but wounded. A gun was found close by him, of which one barrel would appear to have been recently discharged; the other was not discharged, but was loaded with powder only. An inspector of police was sent for, and he cautioned him to be careful in what he said. Notwithstanding that caution, he used the words, "Of course he is dead—he's been teazing me a long while;" and added, "He has made me very nervous for the last month." And upon another occasion he said something to the effect that he understood and wished that Stone was dead.

Mr. Hay, a surgeon of Bridport, made an examination of the body of deceased; then went to the prisoner's house, found his mouth bleeding, his lip blackened, and there was a scorched appearance. The left eyebrow was blackened, and also the skin above the eyebrow. There was a large wound on the forehead, exposing the bone with the edges blackened. The wound was about two inches long, and eleven and a half inches broad. It had ceased bleeding, but there was a pool of blood in the room. His pulse was thick, but feeble. He was much excited. Attended him before a good many years ago. At that time he had his room fastened in with blankets and carpets to keep out the air, quite against my wish. That was about seventeen years ago. He was suffering then from hypochondria, from dyspepsia.

Mr. Coleridge addressed the jury for the prisoner. He said, although the act was done, it was not done by the prisoner when he was in a state of mind that rendered him responsible for it—that although he did the act, it was not the act of his mind, and he was entitled to that protection which the law gives to persons whom it has pleased God in His own mysterious providence to afflict with insanity. An amount of evidence very far short of what is called idiotcy, justifies a jury in saying a man is not guilty. If there is a delusion or a diseased impulse to the mind, connected with the act which has been done, so that the act which has been done is directly the result of the mental disease, the man ceases to be responsible.

Dr. Smith, of Weymouth, from time to time attended Fooks. Saw him first in the year 1846. He was in a state of debility and considerable nervousness—a highly nervous and excited state. He consulted me frequently by letter. Afterwards attended him till within six weeks of this occurrence. On Friday, 29th August, was sent to see him. Found a large wound upon his head. His lips were very much swollen and blackened from powder. He said he supposed he must not have been in his right mind, and he would shoot one hundred men under such provocation. He said he had been traduced, and all had been going wrong for some time.

Dr. Harrington Tuke went to the gaol and had a conversation of three quarters of an hour's duration with the prisoner. "He talked freely enough at first, and although I told him I was a mad doctor, he paid much more attention to the surgeon and deputy-governor than to me. He said his general health was now good; that he had not required medical treatment while in the gaol; that he slept well every night; that everybody was very kind to him, and that he was as comfortable as could be; that he knew the position he was in, and how dreadful it was. I asked him about his first illness. He said he saw Dr. Smith sixteen or eighteen years ago—had seen him at intervals ever since—he had suffered from terrible pains in the head, and was very bad in his inside, too. I told him I thought his inside seemed very well then, and he said I was wrong—that he still had pains in his head, and Dr. Smith told him he had no coat to his stomach at all. He spoke freely about this unhappy attack on Mr. Stone, said he never thought he should have hurt anybody. I asked him if he thought he was deranged at the time. He said, 'Oh, no, no, sir, I beant mad.' I asked him if Stone had injured him in any way. He said, 'No, not exactly that; he was always mocking and scoffing at me, and spreading reports of my character.' I pressed him for an instance, but he would not give one. He said he did not know why he did it, but he knew he did it. At the close of the interview, about half an hour after, I went over this again, and he repeated what he had said. I said, 'You must remember some act;' and Mr. Fooks evidently took great pains, against his own interests, to remember something, and then he said, 'Well, I was once told an acre and a half of my fields had been planted with docks, and sure enough there it was, and Stone might have done it." He didn't shoot him for that, but for taking away his character. He thought Stone might have listened at the windows. He was very unwilling to speak about persons jeering at him ; 'But,' he said, 'I suppose, sir, I must tell you.' He seemed to think he must answer my questions. He said he saw Stone from his window, coming up the road, that he fell all of a daze, that he snatched up a loaded gun, ran down to the door, and discharged it at Mr. Stone. Mr. Goode, the surgeon, asked him if he was aware of the dreadful deed he'd done. He said, 'Lord bless'ee, sir, I thought no more of it than shooting a rabbit.' I then asked him about his attempt at suicide; he became dreadfully agitated, and said, 'I can't bear to talk about it.' He said he put the barrel nearly to his mouth—that he could not kill himself, the gun only containing wadding and powder. He therefore loaded again, and fired at his own head. He could give no reason but that he was 'quite loosed;' he wouldn't have killed himself for all Dorchester. The neighbours used to scoff and jeer at him, but he did not know why, and he sent away two of his men during the haymaking time, because they mocked and scoffed him. Mocking and scoffing he frequently complained of. He said he never threatened suicide, but if Dr. Smith said he did, he supposed he did. He quite knew his position. He thought he had no chance to-morrow. Did not know what defence there could be. I could do nothing for him. He said he wanted his niece to get him a game certificate before she left. I said that was an odd thing for a man who was going to shoot another. He laughed at this, and said 'twas. That others laugh and jeer at them is a common delusion among insane people, and my examination left no doubt on my mind but that he is at this moment of unsound mind; that he has homicidal and suicidal tendencies, which come on periodically—the natural characteristic of the disease. I would also say that the particular form of malady under which he suffers renders nugatory any effort at self-control. The will especially suffers in such cases, and I do not believe him responsible for his actions."

Witness said Esquirol was a great authority in lunacy. A passage from his work, describing the state of a person who acted from uncontrollable impulse, was read, and he subscribed to its accuracy.

Cross-examined by Mr. Collier: Do you adopt the idea of an irresistible tendency to commit homicide?—Witness: I never saw such a case.—Mr. Collier: Then you do not believe this to be a case of irresistible tendency to commit homicide?—Witness: Oh yes, I do, when there is disease of the brain.—Mr. Collier: Do you believe in an irresistible tendency to steal?—Witness: Again I must say, if he has disease of the brain. I should require evidence of a diseased brain.—Mr. Collier: At the same time you consider there is a disease of the brain which develops itself into an inclination to steal?—Witness bowed assent.—That is called *Kleptomania*, is it not?—Witness: That is the scientific term.—Mr. Collier: You are a Kleptomaniac? (laughter). I mean you believe in Kleptomania?—Witness: You have said so, I don't say so at all—a man may be mad, and have an inclination to steal, but there must be disease of the brain.—Mr.

Collier: Then do you believe in a homicidal mania that may be called pure and simple or not?—Witness: I never saw such a case, but the evidence is so irresistible, I must believe it.—Mr. Collier: I understand you to say it is a very common thing for a madman to suppose that persons are jeering at him. I think I might ask you whether that is not a common thing in ordinary life, with those who are not mad?—Witness: I was alluding to a stronger point—a fixed idea that everybody is laughing at you. My opinion of his insanity was partly based on his statement that Stone had annoyed him. I had no means of knowing if it was true.—Mr. Collier: Then if that was founded in truth, your opinion would be altered?—Witness: No, it would not. Supposing it had been proved that Stone had jeered or laughed at him, it would not account for the excessive revenge he took. It would lessen the delusion, but be an exaggeration of the fact, which would amount to insanity.—Mr. Collier: Then do I understand you to lay down the principle that a man can never be so far aggravated as to shoot a man unless he is mad?—Witness: A man in the height of passion might; but a man deliberately to go and shoot another and then himself because he was mocked and jeered, seems to be inconsistent with sanity.—Mr. Collier: You think it would be evidence of insanity for a man to shoot another, unless immediately after the jeers?—Witness: Or unless a man had showed such a disposition to be consistent with his former life.—Mr. Collier: Well, suppose you find that the man was of such a temper that he had declined to be reconciled—that he had thrown a stone at him—would that alter your opinion?—Witness: I should assume the same delusion was still going on. There was more here than jeering—taking away his character, traducing him.—Mr. Collier: Well, supposing it had been shown that he had done so, that would account for it?—Witness: That would account for his anger, but not for his shooting.—Mr. Collier: Then do you think that if a man shoots another for jeering, he must have been under a delusion?—Witness: No; I think you are putting it too strong.—Mr. Collier: You don't go that length?—Witness: No. The witness said he took all the circumstances together, and the circumstances remaining what they are, his opinion was unaltered.

The Judge: Could you say, as a man of science, that a man, who for the last three years had been in the constant use of fire-arms, and never attempted homicide before, is under the influence of a homicidal mania?

Witness: I do not; in this particular case, my lord, it came on in paroxysms. There appear to have been three marked paroxysms; one sixteen years ago, another eight, and another six months. During that time—the last five or six months—I am surprised that he did not kill any one, but before that the exacerbations did not come on.

The Judge: That don't answer my question. Do you, as a man of science, think that you are justified in saying that a man who had used fire-arms, day after day, morning, noon, and night, and has never attempted it before, is under incurable homicidal mania?

Witness: The homicidal mania is not a case that has come much within my experience. I think it to be a means of delusion which were occasionally—only occasionally—beyond his control; and that at this particular time.

The Judge: You have heard all the evidence in this case. As a man of science, from what you have seen of the prisoner and what you have heard of the case, can you undertake to say that when he raised the gun against Mr. Stone, he did not know it would kill him.

Witness: I am sorry to say I think he did know it would kill him, but I think he pulled the trigger against the natural and healthy promptings of the mind, that he could not control himself. I have seen patients of my own exclaim with regret against their tendency to suicide, and immediately after attempt it.

The Judge: From what you have heard of this case and seen, can you, as a professional man, with due regard to the solemnity of your oath, say that, in your opinion, at the time he fired that gun, he did not know that what he was doing was wrong?

Witness: I have the greatest difficulty in answering that question. He certainly knows it is wrong now. But, on my oath, whether he knew it to be right or wrong, he was under an uncontrollable impulse.

The Judge: That is, an homicidal mania beyond his own control?

Witness: Yes.

Mr. Good, surgeon at the gaol, had frequently seen the prisoner since September 4th, and had no opportunity of seeing anything that would justify his saying he is insane.

Conversed with him?—Frequently; did not observe any incoherency; did not see anything that would indicate insanity; had not discovered he suffered under any delusion. My principal study of mania has not been obtained from books but from common sense. When you converse with a man I do not think much professional skill is required. In the course of a long conversation you might arrive at the truth.

Mr. Curme, surgeon, during the absence of Mr. Good, attended the prisoner from September 19th to October 7th. Saw him every day, conversed with him, but not to any great extent. During the whole time he visited him, he did not see any indication to suicide or to homicide.

Mr. Collier replied upon the whole case. The main facts of this case were unfortunately too clear. It was too clear that the prisoner shot the unfortunate man; he was afraid it was too clear, that when he raised the gun to his shoulder he knew he should kill him; that he had a grudge against him of long standing, and that on one occasion he went so far as to throw a stone through a hedge at his cousin. Commenting on the evidence of Dr. Tuke, he hoped that gentleman would acquit him of giving offence if he spoke of him in the terms he himself had used, as the Mad Doctor. Men whose business it is to look out for insanity are apt to discover it where others do not find it to exist, and he had heard that some gentlemen of this class pushed their opinions so far as to believe that there were very few people perfectly sane. Who was likely to have formed the best opinion upon the case—the prison surgeons, who had seen the man for six months, or Dr. Tuke, who saw him for three hours? It is a most dangerous doctrine to society to set up a man's ungovernable passions as a proof of his insanity. It does not do for men who owe an inveterate grudge against their neighbours, instead of endeavouring to smother it, to feed upon and brood over it. It does not do to say he committed murder from an irresistible impulse which he could not control. The object of the administration of the law is to make men control their evil passions.

The Judge commenced by saying, that although some exceptions had been taken by both the learned counsel to the conduct of the case, he himself could not remember anything to have been done that he could wish undone. It appeared to him that they were at that moment in as favourable a position to dispose of the case as it was possible to be in. It was a case, he need not tell them, of the most serious character, and it was a case peculiar for them. They had to decide it, and when he had discharged his duty of reading through the evidence, it would be theirs to say, ay or no, was Daniel Stone wilfully and feloniously murdered by Charles Fooks, of his malice aforethought. Before he went through the evidence, let him state what the law is. He must tell them that the law presumes all homicide to be murder unless the contrary be proved. It was not incumbent on the prosecution (although it had been done most properly in this case) to show malice; it was enough to prove the prisoner had killed a man, leaving to him the duty of excusing or palliating the act. Their whole object ought to be the elucidation of truth. The lives of innocent men were dear to their country; but it was also equally important that murder should not go unpunished. He would now state what the law of insane delusions was. It was not pretended that the prisoner at the bar was generally insane, not contended that his reason was destroyed, or that he had not mind enough to conduct all the ordinary business of life. He was thought sane enough in his parish to be waywarden and overseer, and though not a good accountant, he was trusted by his parish as a man of good sense and ability for such important offices. It was plain by the evidence, that up to the 29th of August he managed his own farm and conducted all the business of life without failing in any respect in intellect. There was not a single witness to show his mind had become so weak that it was easy to overreach him. Now, the law of insane delusions he took to be this: If a man is really under an insane delusion as to the existence of a state of things which, if they did exist, would justify or excuse the act, then he is not punishable for having done it. If, for instance, when the prisoner raised his gun to his shoulder, he believed Stone was about to kill him, then, inasmuch as if Stone had tried to do so, it would have been lawful to resist him, the prisoner would be excused for killing Stone. But if he was under the insane delusion only that he owed an inveterate grudge because he had jeered at him, it would utterly fail to justify his act. It would be for them to say whether such irresistible impulse—such homicidal mania existed in the mind of the prisoner as had been set up. It must be clearly proved to their satisfaction, that at the time the prisoner did the act he was labouring under such a defect of reason as not to know what he was about. The learned Judge then proceeded to review the evidence. Whether he intended to destroy himself or not he thought was quite immaterial. It might on the one hand be evidence of delusion—on the other, of a feeling that he might as well die at once as wait for his execution. With regard to the imputation that common sense is inferior to science, I must say, said the learned Judge, I don't think it is better, although common sense is often informed by science. If common sense is not to decide, you ought not to be in that box; as juries do not pretend, and are not expected to have any scientific knowledge, and after all it is a matter of common sense enlightened by such scientific evidence as the law allows to be laid before you; but still it is for you to decide by your common sense whether his mind was in such a state as not to know he was doing wrong. You are not to be deprived of the exercise of your common sense because a gentleman comes from London and tells you scientific sense. Very often the evidence of scientific gentlemen, particularly of that honourable profession, is but common sense. Referring to the evidence of the delusions of the prisoner, his Lordship said, supposing that Stone was spreading reports traducing his character is not a sufficient excuse for the killing. It is for you to judge whether this man was under the influence of an irresistible influence to kill people. Then, with respect to the evidence of Drs. Good and Curme, it was given quite creditably, and he did not see the slightest disposition on their parts to use one more word against the prisoner than they felt themselves bound to do by the solemn nature of their oaths. His Lordship, in conclusion, said: This is the whole of the evidence for the prosecution, and for the defence. I have done my best to lay it fully and impartially before you. I tell you now, as I told you in the beginning, this is not a question for me to decide, but for you. It is my solemn duty to tell you what the law is upon this point, and your solemn duty, honestly, conscientiously, and firmly to apply that law to the facts of the case, to come to an honest conclusion which you can reflect on with satisfaction, even though it be with regret, to the last days of your lives. I have now to tell you as a matter of law, that although you may be satisfied this man had some insane delusions; yet, if at the time he did this act he knew the nature of that act, and knew what he was doing, and what the consequence would be, and also that it was wrong, then it is your duty to find him guilty; but, if taking into your calm and conscientious consideration all the circumstances of the history of his disease for the last seventeen years, disease in the chest, disease in the head, the frequent expressions of his readiness and willingness to kill any one, you really and seriously think that at the time he pulled the trigger of that gun and shot Stone, he did not know what the effect of what he was doing would be, or what was the nature of his act, or that he was in such a state of mind as to be insane, so that he did not know it was wrong, then it is your duty to find him not guilty. I don't know that I need say anything more, but I beg of you not to hurry to a conclusion, and I am sure you will discuss fully and carefully the case. Do not allow any sense of fatigue or weariness to prevent you giving it the fullest possible investigation, and when you have made up your minds like honest men, be it for the Crown or prisoner, the public will be satisfied with the way you have attended to the case, and you may look back upon it without a feeling of reproach. Consider your verdict.

The jury retired at seven o'clock, and returned a verdict of guilty.

The learned Judge said: I am pained to say that I see no reason for doubting that the jury have come to a correct conclusion, or for doubting that you, under the influence of your bad passions and of your ill-feeling to the unfortunate deceased, whom you sent, without any time to make his peace with God, out of this world, have been guilty of the most heinous offence with which you have been charged. It is my duty, and it is a duty not merely of justice but of charity and kindness to you, to tell you that I cannot hold out to you the smallest hope that your life will be spared.

---

At the Maidstone Assizes, before Mr. Justice Wightman, the young man Burton, eighteen years old, was indicted for the murder of a boy on Chatham Lines last summer. The prisoner, the day after the act, gave himself up to the police. He stated that he had felt an impulse to kill some one; that he had sharpened his knife for the purpose, and went out to find somebody on whom he should use it; that the poor boy was the first person he saw; that he followed him to a convenient place, knocked him down, cut his throat, knelt upon him, and pressed the blood out of him until he was dead. This was the account which the prisoner had himself given of the act, and the only question was as to his accountability for it.

When called upon to plead, he appeared quite to understand the nature of the case, and at once said "Guilty;" and upon the clerk of assize explaining that the charge was that he had wilfully and maliciously murdered the boy, he again said that he desired to plead "Guilty."

Mr. Ribton (counsel for the prisoner) said his delusion was that he desired to be hung; but

The learned Judge, with great firmness of voice and tone, at once said: Let the prisoner understand that the charge is one of wilful murder, and that the plea of "Not guilty" did not mean a denial that he might be guilty of the act, but the effect of it was no more than that he desired to be put upon his trial, and it was far better, for all purposes, that the prisoner should plead "Not guilty," in order thus to be put upon his trial; and (said the learned Judge, with great emphasis) "I warn the prisoner that the plea of 'Guilty' will have no effect whatever upon the Court in averting the sentence of the law, and I advise him, therefore, to retract that plea and plead 'Not guilty.'"

The prisoner, who had listened with apparent intelligence, at once pleaded "Not guilty."

The effect of this, as a comment upon the statement of the prisoner's counsel that he was labouring under insane delusion, was obviously very great.

Mr. Barrow, for the prosecution, adverted to the defence of insanity which he understood was to be set up, as to which it would be for the prisoner's counsel to call evidence to establish it. He would remind the jury that every man was presumed to be sane until the contrary was proved, and that in the circumstances of the case itself there was nothing to indicate the presence of insanity in the mind of the prisoner. On the contrary, he gave the most clear account, not only of the manner in which he had committed the act, but of the feelings under which he had done it, and of the manner in which he had been engaged during the day on which the deed was done.

The prisoner was proved to have said, "I murdered the boy on the Lines;" that he stuck him in the neck, and threw him down, grasped him by the neck, and squeezed him until the blood gushed out of his nose and mouth; that he knelt upon his belly, and trampled upon his face and neck.

All these details were given voluntarily.

The police superintendent stated that when he gave the prisoner the usual caution, he said, "I have made up my mind to tell the truth, and to tell you all about it," and then went on to give a variety of particulars as to the place and mode of murder. On a subsequent occasion he said to the superintendent, "I want to tell you some more particulars."

This was the case for the prosecution.

The prisoner throughout seemed to follow the evidence with perfect intelligence, and as it went on, although at first he seemed indifferent, and now and then smiled at some casual detail, he appeared to be aware of its importance; and when his counsel rose to address the jury, listened to him with earnest attention.

Mr. Ribton addressed the jury at some length on his behalf, not disputing the facts, but enforcing a defence on the ground of insanity. He said the law on the subject was now completely established. There were two cases in particular upon which he relied. One of them was the case of Hatfield, who had fired at George III., and was defended by Mr. Erskine on the ground of insanity. It was proved that he believed he was in communication with the Almighty, and that it was for the welfare of the world that his own life should be sacrificed; and with that view he committed the act in order that he might be executed, going about it with the utmost deliberation. No doubt it was proved that the man had suffered a wound on the head which had caused an injury to the brain. Still it also appeared that he was under the influence of the delusion when he committed the act. The other case was that of Macnaghten who, in 1843, was tried for the murder of Mr. Drummond, and defended by the present Lord Chief-Justice Cockburn. On that occasion Dr. Forbes Winslow gave scientific and speculative evidence on the subject of insanity (never having seen the prisoner before the act), the result of which was that the Attorney-General (Sir William Follett) abandoned the prosecution. It appeared in that case that there was nothing in the prisoner's conduct which could denote the absence of intelligence or sanity, but that there was a conspiracy to injure him, and that he did the act under the influence of this delusion. The speeches of Mr. Erskine and of Mr. Cockburn contained the whole law and science of the subject. Undoubtedly, it was not enough that there should be delusion if the party knew the difference between good and evil or between right and wrong. If, however, that rule were rigidly applied, no one would escape whose reason was not wholly prostrated. The great majority of these unhappy persons were sane enough in their ordinary acts and conduct; but they were under the influence of delusions. Therefore Mr. Erskine had laid it down that delusion was the true character of lunacy. It was true, he admitted, that he must show the relation between the disease and the deed, and said, "I must prove, not only that the prisoner was lunatic (in this sense of the term), but that the act was the immediate offspring of the disease." The test, therefore, was not whether or not the prisoner knew the nature of the act, or knew that it was wrong. So Mr. Cockburn cited many authorities to the like effect:—"The question," said Mr. Cockburn, "is not whether the individual knew that he was killing another, but whether, under a delusion of the mind, he did an act which he would not have done but for that delusion." That was the real and true test; and applying it to the present case, the evidence showed that but for the effect of a delusion the prisoner would not have done the deed. It might be that he knew he was doing what was wrong, but if he did it under the influence of a delusion he was not responsible, for he could no more help his delusion than he could the colour of his eyes or of his hair. The nature of man consisted of various faculties or elements, some moral and some mental, and some might be sound and sane while others were diseased. He cited the resolutions of the Judges (in answer to the House of Lords), agreed to after Macnaghten's case, as to the defence of insanity in criminal cases.

"Assuming that your Lordships' inquiries are confined to those persons who labour under partial delusions only and are not in other respects insane, we are of opinion that, notwithstanding the party did the act complained of with a view, under the influence of insane delusion, of redressing or avenging some supposed grievance or injury, or of producing some public benefit, he is, nevertheless, punishable according to the nature of the crime committed, if he knew at the time of committing such crime that he was acting contrary to law." "That the jury ought to be told in all cases that every man is presumed to be sane, and to possess a sufficient degree of reason to be responsible for his crimes, until the contrary be proved to their satisfaction; and that to establish a defence on the ground of insanity it must be clearly proved that at the time of the committing of the act the party accused was labouring under such a defect of reason from disease of the mind as not to know the nature and quality of the act he was doing, or, if he did know it, that he did not know he was doing wrong. The mode of putting the latter part of the question to the jury on these occasions has generally been, whether the accused at the time of doing the act knew the difference between right and wrong; which mode, though rarely, if ever, leading to any mistake with the jury, is not, we conceive, so accurate when put generally and in the abstract as when put as to the party's own knowledge of right and wrong in respect to the very act with which he is charged; if the accused was conscious that the act was one which he ought not to do, and if that act was contrary to the law of the land; and the usual course, therefore, has been to leave the question to the jury whether the party accused had a sufficient degree of reason to know that he was doing an act that was wrong; and this course we think is correct, accompanied with such observations as the circumstances of each particular case may require."

He went on to argue that the real effect of these answers was, that it was not enough that the accused knew the nature of the act, if he did it under delusions. After having at considerable length dealt with the general question, the learned counsel came to the particular evidence on which he should uphold the defence of insanity. In the first place he should prove that there was insanity in the family, and he cited Taylor's *Medical Jurisprudence* to show that a predisposition to insanity was frequently transmitted from parent to child, especially by the mother. The prisoner's mother had been twice in a lunatic asylum, and though now at home, was under some degree of aberration. A younger brother of the prisoner was nearly lunatic. The prisoner was always eccentric and strange in his conduct, and unlike other boys. He had not merely been eccentric—there were proofs of a depraved and perverted mind. He had been known to kill cats, put them in a pie, and send them to the bakehouse. [The prisoner here smiled, but resumed the aspect of deep attention.] The learned counsel went on to urge the topic of "homicidal mania," and observed that the medical profession did not assent to the doctrine laid down by the Judges on the subject, and held that it was not enough that the criminal knew the act to be wrong, but that it must appear that he had the full control over the faculties of the will. He went on to cite other passages from Dr. Taylor's work to the like effect, describing, as an indication of this species of insanity, a morbid desire to be hanged; and he urged that the prisoner's conduct after the deed evinced the existence of this desire. It was, he urged, latent to the last, and could not be detected as bodily disease might be. Hence it was a matter, necessarily, of medical science and skill, and he was unfortunately unable, on the part of the prisoner, to bring down from London any of the great lights of the profession, and could only call one medical man.

After the evidence of various parties as to peculiarities in the prisoner's conduct and apparent vacancy of mind, a surgeon practising at Chatham was called, and stated that he had attended the prisoner's family, and had sent his mother on two occasions to a lunatic asylum. She was low and desponding, and attempted suicide. The prisoner's brother, too, was of weak intellect. He was peculiar-looking and was dissipated. He answered in monosyllables, and used silly expressions. As to hereditary insanity, he agreed with Dr. Taylor, and he believed his to be the opinion of all eminent writers. So, also, he agreed with Dr. Taylor as to "homicidal mania." All these aberrations had their origin in functional organic derangement of the brain, which could not always be detected even in dissection after death, and which was necessarily matter of speculative opinion. On two occasions he had attended the prisoner himself, and believed he was labouring under what in the profession would be considered a "moral insanity"—that is, he knows perfectly well what he is doing, but has no control over himself.

Mr. Ribton.—I presume that by moral insanity you mean a disease of the moral feelings as distinguished from the intellectual? By the moral feelings I mean the propensities. May they be diseased while the intellectual faculties are sound?

Witness.—Yes; as a matter of science, I should say so.

The learned Judge.—As a matter of science! It is hardly necessary to have recourse to science to learn that the moral faculties may be diseased while the intellectual faculties are sound. No doubt there may be a disease of the moral feelings while the man is in perfect possession of his senses.

Mr. Ribton said he desired to show a moral disease distinct from depravity.

The witness went on to state that, in his opinion, it was reasonable to believe that there must in such a case be some derangement of the brain—some deviation from the normal condition of the brain. Having heard the evidence in this case, was of that opinion as regarded the prisoner.

The learned Judge.—Do you consider it a mark of moral disease that he should eat a piece of a cat?

Mr. Ribton suggested that the question was, in what sense the word "moral" was used.

The learned Judge observed that the difficulty was to know in what sense the word was used.

The witness said a man might be able to work out a proposition in *Euclid*, and yet might eat a piece of a cat. The intellectual functions might be unimpaired, whilst the moral functions were deranged.

In cross-examination, the witness stated that he believed the prisoner knew what he was doing, but that an impulse came upon him which he could not control; and he cited and adopted an opinion of Dr. Forbes Winslow's, that no man could commit suicide in a state of sanity. He believed that the prisoner had no proper control over his actions. He had a knowledge of right and wrong, but could not control his actions.

Witnesses were then called on the part of the prosecution to rebut the defence of insanity thus set up.

Mr. Joy, the surgeon of the county prison, stated that he had observed the prisoner ever since he was put in confinement, and in his opinion he was perfectly sane, nor did he ever observe that he was under any delusion. The prisoner had certainly said that justice would not be done if he were not executed. When pressed as to whether he had not heard him express a desire to be hanged, he said that he had not himself heard him say so. When asked: Suppose a man with a desire to be hanged, committing a homicide with that object, would that be a mark of insanity? The witness said he thought it would be, but that he had not heard or read of any case of that kind, except that of Macnaghten.

Another of the medical attendants was called, and gave similar evidence as to his belief in the prisoner's sanity.

Mr. Ribton, in summing up the evidence for the defence, insisted that the prisoner had a vehement desire to be hanged, and urged that this was the strongest proof of insanity.

Mr. Barrow replied on the part of the prosecution. The question was whether the prisoner, at the time of the act he committed, was in such a state of mind as not to be responsible for his actions. The defence set up really came to this—that a man was not responsible for his actions if he was not in full possession of the moral or intellectual faculties. But that was not enough, and the rule of law, as laid down by the Judges, was that the man was responsible if he knew right from wrong, and knew that he was committing an offence against the laws of God and nature. The evidence in this case did not show that the prisoner did not know he was doing wrong. In the cases cited there was evidence of delusions leading to homicide. Here, on the contrary, there was no evidence of any delusions at all. The argument for the defence came to this—that a defect in the moral faculties was tantamount to insanity, and went wholly to confound depravity with insanity. Why, all criminality more or less must involve some defect or alienation of the moral faculties. So, according to this theory of insanity, there could be no such thing as criminality, or crime would easily secure a perfect impunity.

Mr. Justice Wightman, summing up the case, said the jury were engaged in one of the most momentous inquiries which could occupy the minds of men, and which was one of the deepest importance to the interests of society. There was no doubt about the act; the only question was whether the prisoner at the time he committed it was in such a state of mind as not to be responsible for it. The prisoner's account of it was that he had done it from a morbid feeling; that he was tired of life, and wished to be rid of it. No doubt prisoners had been acquitted of murder on the ground of insanity; but the question was, what were the cases in which men were to be absolved from responsibility on that ground. Hatfield's case differed from the present, for there wounds had been received on the head which were proved to have injured the brain. In the more recent case of Macnaghten, the Judges had laid down the rule to be that there must, to raise the defence, be a defect of reason from disease of the mind, so as that the person did not know the nature and quality of the act he committed, or did not know whether it was right or wrong. Now, to apply this rule to the present case would be the duty of the jury. It was not mere eccentricity of conduct which made a man legally irresponsible for his acts. The medical man called for the defence defined homicidal mania to be a propensity to kill, and described a moral insanity under which a man, perfectly aware that it was wrong to do so, killed another under an uncontrollable impulse. This would appear to be a most dangerous doctrine, and fatal to the interests of society and the security of life. Surely such a theory was as contrary to common sense as it undoubtedly was to law. The rule, as laid down by the Judges, was quite inconsistent with such a view, for it was that a man was responsible for his actions if he knew the difference between right and wrong. It was urged that the prisoner did the act to be hanged, and so was under an insane delusion; but what delusion was he under? So far from it, it showed that he was quite conscious of the nature of the act and of its consequences. He was supposed to desire to be hanged, and in order to attain the object, committed murder. That might show a morbid state of mind, but not delusion. Homicidal mania, again, showed no delusion. It merely showed a morbid desire for blood. Delusion meant the belief in what did not exist. The question for the jury was whether the prisoner at the time he committed the act was labouring under such a species of insanity as to be unaware of the nature, the character, or the consequences of the act he committed. In other words, whether he was incapable of knowing that what he did was wrong. If so, they should acquit him; if otherwise, they should find a verdict of guilty.

The jury pronounced a verdict of GUILTY.

In passing sentence, the learned Judge addressed the prisoner in a very emphatic tone in these terms: The jury, who are the proper judges of the fact, and who have, I am sure, attended most carefully to the evidence, have come to the conclusion that you are guilty of a murder more barbarous and inhuman than any which has come under my cognizance during a judicial experience of upwards of twenty years. The victim of your crime was a poor little boy between nine and ten, described by the witnesses as an intelligent and good little boy, who had never, in any way whatever, given you the slightest offence, and whom you murdered in a manner and with circumstances so cruel that I could not trust myself to dwell upon its shocking details. It is said that you laboured under a morbid desire to die by the hands of justice, and that for that purpose you committed the murder. This morbid desire to part with your own life can hardly be called a "delusion;" and, indeed, the consciousness on your part that you could effect your purpose by designedly depriving another of life, for which you would have to suffer, as you know, the punishment due to the greatest of crimes, shows that you were perfectly able to understand the nature and consequences of the act which you were committing, and that you knew it was a crime for which, by law, the penalty was capital. This was, in truth, a further and, I may say, a deep aggravation of the crime; for you designedly intended to compass your own death by the murder of another. For such a crime as yours there can be no hope of mercy in this world, even if you now wished it. Let me, then, earnestly entreat you, by sincere repentance for this your terrific crime, to endeavour to obtain that mercy beyond the grave which you cannot hope for upon earth. The duty only remains for me to pass upon you the sentence of the law. The learned Judge then passed sentence of death in the usual form.

The prisoner, who had listened throughout with serious attention, assumed at the close the air of callous indifference, which seemed his habitual expression; and after a pause of a few moments, in which a sense of awe seemed to struggle with a desire to take a tone of bravado, he evidently almost forced himself to brazen it out, and, with an impudent smile, said, "Thank you, my Lord," and went quickly down the dock, followed by an audible murmur and almost a cry of horror from a densely crowded audience.

---

